# White and red plaques of the labia and inguinal folds

**DOI:** 10.1097/JW9.0000000000000183

**Published:** 2024-11-01

**Authors:** Ana M. Aragon Sierra, David J. DiCaudo, Leah A. Swanson

**Affiliations:** a Mayo Clinic Alix School of Medicine, Scottsdale, Arizona; b Department of Dermatology, Mayo Clinic, Scottsdale, Arizona; c Department of Laboratory Medicine and Pathology, Mayo Clinic, Scottsdale, Arizona

**Keywords:** granular parakeratosis, vulvar dermatosis

**What is known about this subject in regard to women and their families?**

- Granular parakeratosis is a rare reactive dermatosis that presents as pruritic red/brown plaques.
- It most commonly affects women over age 40 manifesting in intertriginous sites, with up to a third of cases presenting in the groin.

**What is new from this article as messages for women and their families?**

- Granular parakeratosis can manifest in an atypical presentation as white plaques distributed on the vulva.
- This article draws attention to the unique and multiple potential contactants, friction/maceration, and altered microbiome that could contribute to granular parakeratosis of the vulva.

## Case summary

**Fig. 1.**
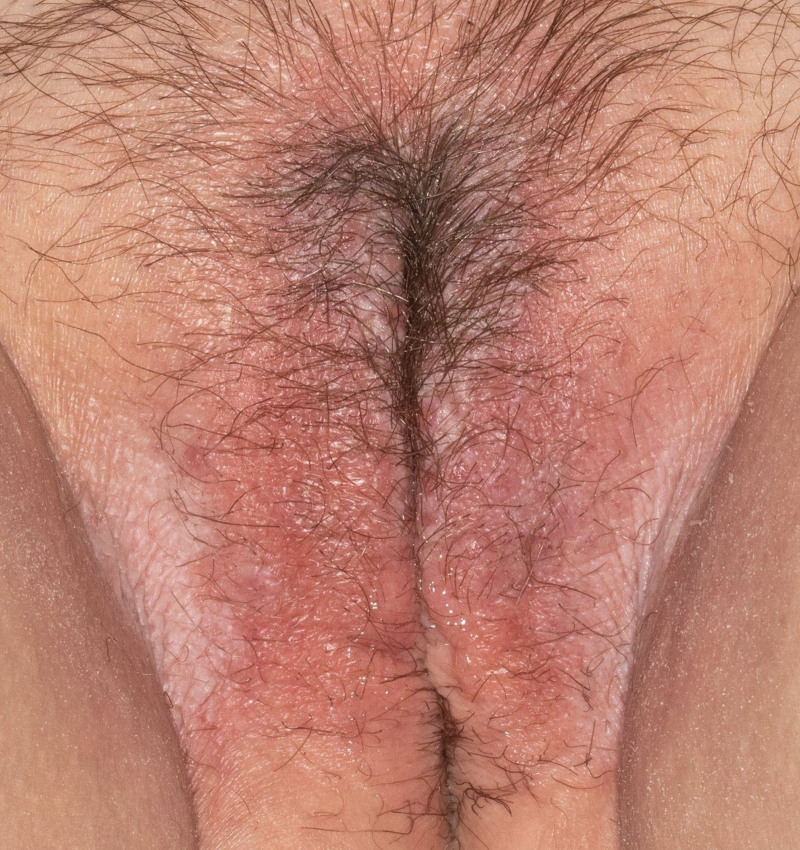
Clinical photograph of red plaques across the labia majora and white lichenified plaques along the medial labia majora and inguinal folds.

**Fig. 2.**
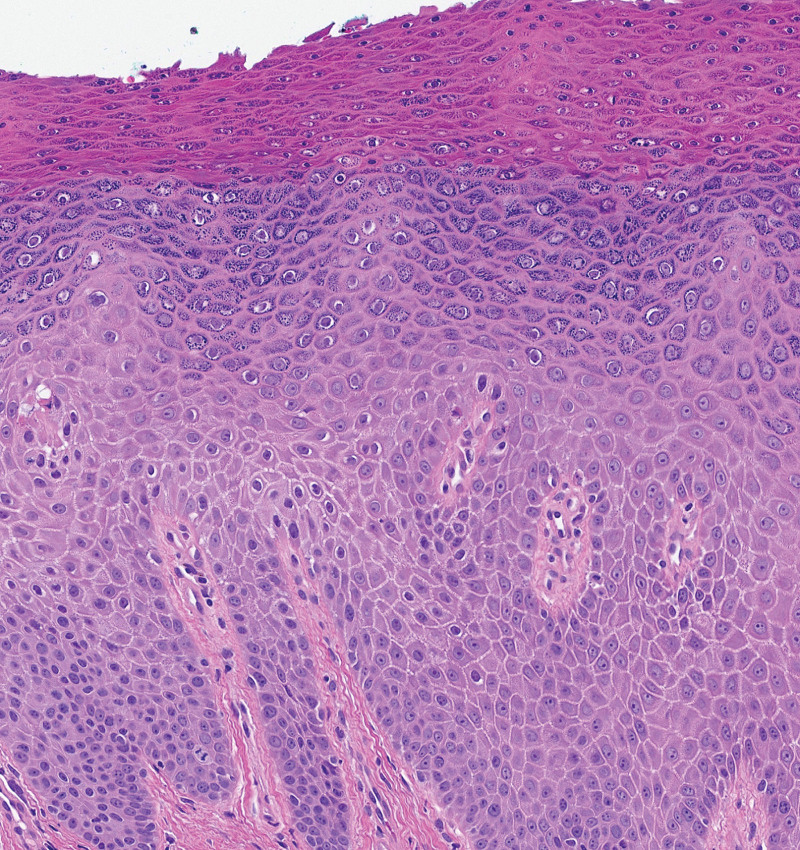
Histopathology of biopsy from white plaque demonstrating both spongiosis and psoriasiform hyperplasia of the epidermis with hypergranulosis and retained keratohyalin granules within stratum corneum (hematoxylin–eosin, original magnification ×100).

A woman in her 40s with a past medical history of allergic rhinitis, asthma, and childhood eczema was referred to dermatology for chronic vulvar pruritus and vulvar rash refractory to treatment. The patient had failed topical corticosteroids, oral prednisone, topical estradiol, and oral gabapentin. On exam, there were bright red lichenified plaques diffusely involving the labia majora with more discreet white papules on the medial aspect and white lichenified plaques extending from the lateral labia majora to the inguinal folds bilaterally (Fig. 1). Punch biopsy was obtained from a white plaque on the labium majus with histopathologic features shown below (Fig. 2).

**Question 1**

**What is your diagnosis?**

A. Tinea cruris
B. Psoriasis
C. Granular parakeratosis
D. Lichen sclerosus
E. Candidiasis

**Correct answer:** C. Granular parakeratosis. On histopathology, there is spongiotic and psoriasiform dermatitis with hypergranulosis and retained keratohyalin granules within the stratum corneum. These histopathologic findings are consistent with granular parakeratosis (GP) associated with a chronic spongiotic process. The retained keratohyalin granules in the stratum corneum are distinct from the hyphal and yeast forms observed in tinea cruris and candidiasis, respectively. Although psoriasiform hyperplasia is observed, the granular layer is accentuated and the spongiosis is greater than typical for psoriasis. In lichen sclerosus, the epidermis is often atrophic with underlying homogenized dermal collagen and a lichenoid band of inflammation.

## Discussion

GP is an uncommon, idiopathic reactive process of the skin. Most often, it presents as red to brown hyperkeratotic papules or plaques distributed bilaterally in intertriginous areas.^1,2^ Although the axilla is most commonly affected, a systemic review found that a third of cases involved the groin.^1^ Vulvar dermatoses can develop a whitish color secondary to moisture, and a case report has confirmed that GP can have a gray-white appearance within the groin.^3^ GP is reported most often in women over the age of 40, although men and women of all ages may be affected.^1,2^

GP was initially thought to be secondary to a contact dermatitis to deodorants and antiperspirants.^1^ However, later a broader range of contactants were implicated and occlusion was also thought to play a major role in pathogenesis.^1,2^ The most commonly implicated topical agents include zinc oxide, deodorant/antiperspirant, and benzalkonium chloride.^1^ Some believe that disorders of cornification are responsible for GP development, but current data supports a combination of an underlying predisposition and exposure to irritants, allergens, friction/maceration, and altered microbiome that culminate in GP.^1,2^

As the etiology of GP is complex, treatments are varied. Many cases resolve with the removal of the external trigger/irritant along with topical corticosteroids.^3^ Other reported treatments include topical vitamin D analogs, calcineurin inhibitors, antibiotics, antifungals, ammonium lactate, topical and oral retinoids, botulinum toxin injection, cryotherapy, surgery, and neodymium-doped yttrium aluminum garnet combined with CO_2_ fractional laser.^1–3^

**Question 2**

**How is the diagnosis of GP made on histopathology?**

A. Psoriasiform hyperplasia
B. Subacute spongiosis
C. Parakeratosis and hypogranulosis alternating with orthokeratosis and hypergranulosis
D. Retained keratohyalin granules within the stratum corneum

**Correct answer:** D. Retained keratohyalin granules within the stratum corneum on histopathology. GP is defined by retained keratohyalin granules within the stratum corneum. Retained keratohyalin granules of the stratum corneum may coexist with psoriasiform and spongiotic histopathologic patterns; however, psoriasiform hyperplasia and subacute spongiosis are not distinguishing features of GP.^2,3^ Parakeratosis and hypogranulosis alternating with orthokeratosis and hypergranulosis is observed in inflammatory linear verrucous epidermal nevi.

**Question 3**

**Which of the following has not been implicated in the multifactorial pathogenesis of GP?**

A. Altered microbiome
B. Autoimmunity
C. Irritant exposure
D. Allergen exposure
E. Occlusion and friction

**Correct answer:** B. Autoimmunity. GP is best considered a reactive change, rather than a distinct disease, which manifests from various but interconnected causes in perhaps a predisposed individual. The most frequently affected sites are intertriginous and occluded. These sites are exposed to friction and sweat, which results in irritation and/or maceration disturbing the skin barrier. A disrupted barrier increases susceptibility to allergic contact dermatitis and overgrowth of normal flora. These factors culminate in the skin’s reactive retention of keratohyalin granules within the stratum corneum. Autoimmunity is not implicated in the pathogenesis of GP.

## Conflicts of interest

None.

## Funding

None.

## Study approval

N/A

## Author contributions

AMAS, DJD, and LAS: Participated in the writing of the paper.

## Patient consent

Informed, written consent was received from all patients for whom photographs are present in the manuscript.
